# Understanding *Pseudomonas aeruginosa* Biofilms: Quorum Sensing, c-di-GMP Signaling, and Emerging Antibiofilm Approaches

**DOI:** 10.3390/microorganisms14010109

**Published:** 2026-01-04

**Authors:** Ayman Elbehiry, Eman Marzouk, Husam M. Edrees, Mai Ibrahem, Safiyah Alzahrani, Sulaiman Anagreyyah, Hussain Abualola, Abdulaziz Alghamdi, Ahmed Alzahrani, Mahmoud Jaber, Akram Abu-Okail

**Affiliations:** 1 Department of Public Health, College of Applied Medical Sciences, Qassim University, P.O. Box 6666, Buraydah 51452, Saudi Arabia; e.marzouk@qu.edu.sa (E.M.); mf.jaber@qu.edu.sa (M.J.); 2 Department of Physiology, Faculty of Medicine, University of Tabuk, Tabuk 74191, Saudi Arabia; hedrees@ut.edu.sa; 3 Department of Public Health, College of Applied Medical Science, King Khalid University, Abha 61421, Saudi Arabia; 4 Department of Basic Medical Sciences, College of Applied Medical Sciences, King Khalid University, Khamis Mishit 61421, Saudi Arabia; 5 Family Medicine Department, King Fahad Armed Hospital, Jeddah 23311, Saudi Arabia; 6 Home Health Care Department, King Fahad Armed Forces Hospital, Jeddah 23311, Saudi Arabia; 7 Academic Affairs Department, Prince Sultan Military College for Health Sciences, Dhahran 34465, Saudi Arabia; 8 Department of Pathology and Laboratory Diagnosis, College of Veterinary Medicine, Qassim University, Buraydah 51452, Saudi Arabia

**Keywords:** *Pseudomonas aeruginosa*, biofilm, quorum sensing, c-di-GMP signaling, antimicrobial resistance, nanoparticles, nitric oxide dispersal, public health

## Abstract

*Pseudomonas aeruginosa* (*P. aeruginosa*) forms biofilms that are difficult to eliminate. The matrix protects the cells, efflux pumps reduce intracellular drug levels, and dormant subpopulations survive treatment. Routine minimum inhibitory concentration (MIC) testing does not account for these features, which helps explain why infections often continue even when therapy appears appropriate. This review describes how quorum-sensing (QS) and cyclic di-guanosine monophosphate (c-di-GMP) regulate matrix production, efflux activity, and dormancy within *P. aeruginosa* biofilms. Important matrix components, including Psl, Pel, alginate, and extracellular DNA, slow the movement of antimicrobial agents. Regulatory proteins such as sagS and brlR increase the activity of the MexAB-OprM and MexEF-OprN efflux systems, further reducing intracellular drug concentrations. Oxygen and nutrient limitation promote persister cells and viable but nonculturable cells, with both having the ability to survive antibiotic levels that would normally be lethal. These defenses explain the gap between MIC values and biofilm-specific measurements, such as the minimum biofilm inhibitory concentration and the minimum biofilm eradication concentration. This review also summarizes emerging antibiofilm strategies. These include QS inhibitors, compounds that lower c-di-GMP, such as nitric oxide donors, nanoparticles, depolymerases, bacteriophages, and therapies that are directed at host targets. Modern diagnostic tools, such as confocal laser scanning microscopy, optical coherence tomography, and Raman spectroscopy, improve detection and guide treatment planning. A staged therapeutic approach is presented that begins with the dispersal or loosening of the matrix, continues with targeted antibiotics, and concludes with support for immune clearance. Viewing these strategies within a One Health framework highlights the role of biofilms in clinical disease and in environmental reservoirs and supports more effective surveillance and prevention.

## 1. Introduction

Biofilms represent a dynamic bacterial lifestyle rather than a fixed state. Bacterial cells coordinate behavior, communicate, and embed themselves in a self-produced extracellular polymeric substance (EPS) matrix that reshapes community function [1]. This collective mode of growth gives bacteria a high level of tolerance to antibiotics and host immunity, which explains the persistent nature of biofilm-related infections in clinical practice [2].

In patients with cystic fibrosis (CF), *Pseudomonas aeruginosa* (*P. aeruginosa*) establishes chronic airway infections that are dominated by biofilm growth. Mucoid conversion and increased alginate production alter the matrix and strengthen the chronic state of infection [3,4]. Experimental and clinical studies show that CF-adapted airway biofilms display elevated tolerance to several classes of antibiotics, which links biofilm behavior to progressive lung disease [5,6]. *P. aeruginosa* also forms invasive biofilms in burn wounds, and animal models reproduce the deep tissue architecture seen in human burns [7,8]. On urinary catheters, surface-attached biofilms lead to catheter-associated urinary tract infections (CAUTI) and often limit the effect of antimicrobial therapy [9]. In ventilator circuits, biofilms that form inside endotracheal tubes act as a source for ventilator-associated pneumonia (VAP) and allow bacterial migration into the lower airways [10,11].

*P. aeruginosa* serves as a model organism for understanding biofilm biology because reference strains PAO1 and PA14 are genetically tractable, well characterized, and show distinct virulence features [12]. The PAO1 genome, which measures about 6.3 megabases, revealed broad regulatory and metabolic capacity that supports environmental adaptability and intrinsic drug resistance [13]. This genomic foundation allows systems-based studies of biofilm control. Comparative analyses highlight the important differences between PAO1 and PA14 and help to distinguish conserved biofilm traits from strain-specific behavior [14]. Alongside genomic work, integrated in vitro, in vivo, and ex vivo model systems now allow a detailed investigation of *P. aeruginosa* biofilms under clinically relevant conditions [15].

A hierarchical quorum-sensing (QS) network that includes the Las, Rhl, and *Pseudomonas* quinolone signal (PQS) systems, with added inputs such as the integrated QS signal, is central to the resilience of *P. aeruginosa* biofilms [16,17]. These systems regulate many of the genes that are responsible for virulence, metabolism, and community behavior that support biofilm development and maintenance [16,17]. In parallel, the second messenger cyclic di-guanosine monophosphate (c-di-GMP) coordinates the transition from motile to sessile growth, guides surface attachment, and promotes matrix production. These activities are shaped by the opposing functions of diguanylate cyclases (DGCs) and phosphodiesterases (PDEs) [18,19]. Beyond early development, c-di-GMP sets the physiological state of biofilm communities and strongly influences metabolic activity in multiple experimental models [20]. Nitric oxide (NO)-induced dispersal depends on the activation of PDEs and a rapid decrease in intracellular c-di-GMP, which links environmental cues to biofilm destabilization [21,22].

Given the clinical threat of multidrug-resistant (MDR) *P. aeruginosa* and the complexity of its regulatory networks, it is essential to understand how QS and c-di-GMP intersect to control matrix production, persistence, and dispersal. This review maps the regulatory circuitry that supports biofilm resilience, explains how these signals shape virulence and antimicrobial tolerance, and evaluates translational strategies that target these systems. These strategies include QS inhibitors, c-di-GMP modulators, dispersal inducers, nanoparticle (NP)-based delivery systems, and bacteriophage-derived enzymes that can reprogram or disrupt biofilm behavior in chronic infections.

## 2. Architectural and Molecular Foundations of *P. aeruginosa* Biofilms

### 2.1. Staged Development: Attachment, Maturation, and Dispersal (pel, psl, algD, sadB, and bdlA)

*P. aeruginosa* follows a stepwise developmental program that includes reversible attachment, irreversible attachment, microcolony formation, three-dimensional maturation, and active dispersal to new sites of infection [23,24]. Irreversible attachment represents a true commitment checkpoint. Loss of *sadB* locks cells into a surface-defective, hyperswarming state and identifies *sadB* as a gatekeeper that also acts as a posttranslational modulator of AmrZ [25,26,27].

**Figure 1:**
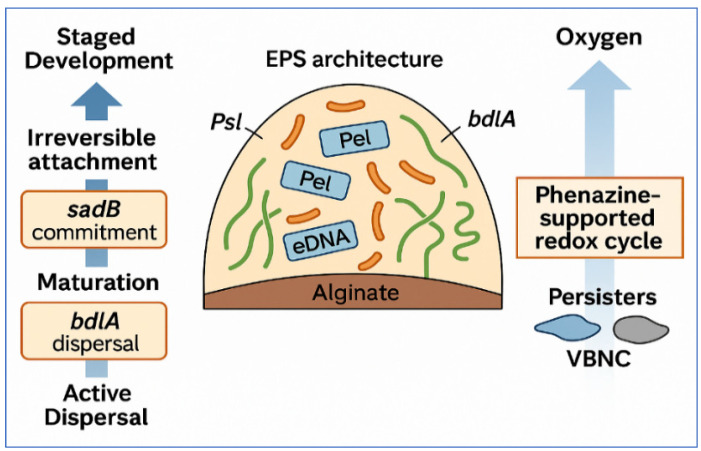
The structural code of *P. aeruginosa* biofilms. A conceptual overview that summarizes how biofilms form, strengthen, and disperse. The panel on the left presents the staged progression from reversible attachment to maturation and active dispersal. These stages are guided by genetic checkpoints that include *sadB*, *pel*, *psl*, *algD*, and *bdlA*. The central panel illustrates EPS architecture, which contains Psl that mediates cell-to-cell and cell-to-surface adhesion, Pel that contributes to cohesion and tolerance, alginate that forms the mucoid matrix, CdrA that crosslinks with Psl, and eDNA that provides mechanical reinforcement. The panel on the right shows how oxygen and nutrient gradients create stratified physiology. These gradients support phenazine-linked redox activity and favor the development of drug-tolerant persister and VBNC subpopulations.

Dispersal is an actively controlled process that requires BdlA, a chemotaxis-like regulator whose phosphorylation state integrates environmental cues with c-di-GMP signaling [28,29,30,31]. Genetic and biochemical studies place BdlA together with partners such as DipA at the center of the dispersal switch [32,33,34,35]. Figure 1 summarizes this trajectory and highlights key genetic checkpoints (*sadB*, *pel*, *psl*, *algD*, and *bdlA*), EPS architecture components (Psl, Pel, alginate, CdrA, and eDNA), and oxygen gradients that support phenazine-driven redox cycling and the emergence of persister and viable but nonculturable (VBNC) subpopulations.

### 2.2. Matrix Composition and Mechanics: Polysaccharides, Proteins, eDNA, and Host Inputs (NETs, Mucins)

The EPS matrix functions as a composite material whose mechanical properties and signaling capacity arise from interactions among polysaccharides, proteins, and DNA. Psl provides architectural stability and binds the lectin LecB, which helps retain cells and EPS within the biofilm [29,36,37]. Pel is a cationic polymer with a wide genetic distribution and enhances both cohesion and tolerance to antimicrobial agents [29,36,37]. Alginate that is synthesized through *algD* reshapes CF biofilms and modifies drug responses, even though many nonmucoid *P. aeruginosa* biofilms form in its absence [31,38].

Matrix-associated proteins further strengthen and specialize the structure. The c-di-GMP-regulated adhesin CdrA crosslinks with Psl to promote aggregation and support robust biofilm formation [39,40]. eDNA acts as a load-bearing scaffold that interacts with Psl to create a skeletal lattice within the matrix. Treatment with DNase weakens this scaffold and improves antimicrobial penetration, particularly in early-stage biofilms [41,42]. Host-derived components are integral to matrix organization. Neutrophil extracellular traps (NETs) can surround *P. aeruginosa* communities in dense DNA-rich barriers, while airway mucins reshape the architecture of the biofilm. Depending on the local environment, mucins can stabilize tolerant aggregates or instead promote dispersal [43,44,45].

### 2.3. Spatial and Metabolic Heterogeneity: Oxygen and Nutrient Gradients, Persisters, and VBNC Cells

Steep gradients of oxygen and nutrients stratify *P. aeruginosa* biofilms and generate layers that differ in gene expression patterns, redox programs, and local growth rates [46,47]. In hypoxic or anoxic regions, endogenous phenazines shuttle electrons to sustain adenosine triphosphate (ATP) production. This redox cycling supports fitness under low oxygen conditions and contributes directly to antibiotic resistance [48,49,50]. This physical and chemical heterogeneity promotes phenotypic diversity within the biofilm. Drug-tolerant persister cells arise as dormant but reversible subpopulations and are repeatedly associated with treatment failure and infection relapse [51,52,53].

Other subpopulations can enter a VBNC state in response to disinfectants or antibiotic stress, as demonstrated in chlorine-exposed and catheter-associated biofilms [54,55,56]. Single-cell- and spectroscopy-based studies reveal that VBNC cells display distinct metabolic signatures when compared with neighboring culturable cells. Emerging single-cell transcriptomic maps further refine this view and show widespread transcriptional diversification within biofilms. These studies demonstrate a global metabolic downshift across biofilm cells when compared with planktonic populations [57].

## 3. Quorum Networks: The Social Intelligence of Biofilms

### 3.1. Hierarchical QS Systems Involving las, rhl, pqs, and iqs and Their Cross-Regulation

*P. aeruginosa* relies on two acyl homoserine lactone circuits, known as *las* and *rhl*, and on a quinolone-based circuit that is known as *pqs*. These systems also include an *iqs* module that links nutrient stress to QS activity. In the classical model, LasR activates both *rhl* and *pqs*, while PqsR controls the Pseudomonas quinolone signal and the related metabolite HHQ. These signals feed back into QS activity and guide biofilm programs [16].

The order of Las, Rhl, and Pqs activity is flexible. Many *lasR* variants maintain QS-controlled traits through alternative pathways [58,59]. Under phosphate limitation, PAO1 can shift to a state that does not depend on LasR. In this setting, RhlR dominates and represses *pqsABCDE*, which changes the hierarchy of the system [60]. Phosphate stress activates the *iqs* module. Under normal conditions, *iqs* is under Las control, but phosphate scarcity leads to PhoB-driven *iqs* upregulation and strengthens QS and virulence outputs [61]. Nutritional conditions also influence the relative activity of *las*, *rhl*, and *pqs*, which creates a context-dependent hierarchy [62].

Clinical isolates show similar plasticity. *lasR* variants are common in CF airways and can maintain or restructure QS-controlled pathways, which supports chronic infection [59,63]. A major mechanism behind this flexibility is the partnership between RhlR and PqsE. Structural and functional studies show that PqsE enhances RhlR-dependent transcription. This activity can reduce the need for the native Rhl autoinducer and preserve RhlR-driven behavior [64,65,66]. Recent work indicates that the PqsE and RhlR interactions fine-tune the RhlR output to match environmental cues [67]. The *las*, *rhl*, *pqs*, and *iqs* modules form a network that adapts to nutrient changes and stress. This network contains regulatory handoffs that can be targeted, including RhlR dominance under phosphate limitation and the RhlR and PqsE interaction [16,60,61,64].

### 3.2. Molecular Integration Between QS and c-di-GMP

QS operates within a broader group of second messenger and global control systems that include c-di-GMP signaling, the Gac and Rsm pathways, and the cyclic adenosine monophosphate (cAMP) Vfr system. These systems translate cell density information into motility changes, matrix production, and chronic phenotypes [68]. LasR and RhlR activate matrix-related loci. Evidence from PAO1 shows that *psl* is under QS control, and earlier studies link QS activity to *pel*. Together, these findings place Las and Rhl at the upper level of matrix regulation [69,70].

In parallel, c-di-GMP determines the shift from motile to sessile behavior. High c-di-GMP promotes irreversible attachment and EPS synthesis, while low c-di-GMP supports motility and dispersal. These shifts influence QS-controlled outputs such as rhamnolipid production [18,71,72]. At the core of the QS network, RhlR partners with PqsE to modify transcriptional programs. Structural work identifies a targetable interface between RhlR and PqsE [64,65,73].

Furthermore, c-di-GMP also maintains mucoid behavior. The binding of c-di-GMP to the PilZ domain protein Alg44 is essential for alginate polymerization. In addition, AlgU, which is released during MucA loss, drives *algD* expression and connects with QS to consolidate chronic traits [74,75,76,77]. Higher-level regulators, such as Gac and Rsm small RNAs and the cAMP Vfr axis, interact with DGCs and PDEs, including GcbA, to influence surface commitment and matrix output [78,79,80]. The overall conclusion is that QS and c-di-GMP form a coupled system that stabilizes matrix-rich biofilms, yet it preserves the capacity for motility and dispersal when conditions change [65,68,69,70,75].

### 3.3. QS-Controlled Virulence Portfolio

Across strains, QS controls a broad set of virulence traits that include secreted enzymes, surfactants, redox-active metabolites, and toxins. These traits strengthen biofilms and reduce the effectiveness of therapy. Las, Rhl, and Pqs regulate hundreds of genes, among them being LasB elastase, rhamnolipids that arise from *rhlAB*, several phenazines such as pyocyanin, hydrogen cyanide, and secretion systems that influence tissue injury, motility, and tolerance [81].

The *pqs* module shapes matrix behavior and dispersal. Under PqsR control, PQS and HHQ promote eDNA release through controlled lysis and prophage activation. Loss of *pqsL* increases HHQ levels and autolysis, which increases eDNA in early biofilms [82]. PQS also promotes the formation of outer membrane vesicles that carry eDNA and enzymes and assist in dispersion [83,84].

Phenazines, such as pyocyanin and HQNO, influence redox pathways and interactions between species. HQNO suppresses respiration in *Staphylococcus aureus* (*S. aureus*) and selects small-colony variants that rely on fermentative metabolism. These patterns involve sigma factor B and SrrAB and are common in CF airways [85,86,87,88,89]. Many QS-controlled traits are targetable. Natural compounds and synthetic inhibitors directed at RhlR or PqsR can reduce elastase, rhamnolipids, pyocyanin, and biofilm formation. Some of these inhibitors also modify c-di-GMP levels, which highlights cross-node intervention points [90,91,92].

### 3.4. Polymicrobial and Host Interactions

QS signals shape interactions within mixed communities that are often found in chronic airway and wound infections. HQNO limits respiration in *S. aureus*, reduces ATP levels, and selects fermentative small-colony variants. These responses appear in coculture systems, animal models, and clinical settings and involve SrrAB and SigB [86,93,94,95]. Outcomes depend on context. Oxygen availability affects HQNO production and activity, which determines whether the interaction results in competition or coexistence. Anaerobic conditions and media that mimic mucus can favor mixed microcolonies and isolate specific interactions [96,97].

Beyond quinolones, *P. aeruginosa* produces pyocyanin and the staphylolytic protease LasA. These factors impair *S. aureus* respiration or damage the cell wall, which shifts competitive balance within polymicrobial biofilms [98,99,100]. These exoproducts help explain why *P. aeruginosa* often suppresses or displaces *S. aureus*, but can also coexist with it under certain environmental constraints [101].

Table 1 summarizes the QS modules and their connections to global regulatory systems that include c-di-GMP, Gac and Rsm, and AlgU. The table lists the signals, the synthase and receptor pairs, the major regulatory interactions, the principal outputs, the environmental factors that influence each system, and the main targets described in the literature.

## 4. c-di-GMP Dynamics: The Molecular Switchboard of Biofilm Formation

### 4.1. Synthesis and Degradation Enzymes Involving DGCs and PDEs

In *Pseudomonas aeruginosa*, intracellular c-di-GMP is controlled by a large set of diguanylate cyclases (DGCs) that contain GGDEF domains and by phosphodiesterases (PDEs) that contain EAL or histidine aspartate glycine tyrosine proline domains. Genome-wide analyses of strain PAO1 showed that *P. aeruginosa* encodes approximately 41 proteins involved in c-di-GMP turnover. These include about 17 GGDEF-only proteins, 5 EAL-only proteins, and 16 proteins that carry both GGDEF and EAL domains, with additional HD-GYP proteins also annotated [18]. These opposing enzyme families shift physiology between motile states that require low c-di-GMP and matrix-producing states that require high c-di-GMP. This second messenger is central to biofilm initiation, maturation, and dispersal in PAO1 and PA14 [18,108,109].

The Wsp sensory system translates surface contact into increased c-di-GMP. Activation of WspA through WspE leads to phosphorylation of WspR. Phosphorylated WspR forms clusters that strengthen DGC activity and promote biofilm formation. Fluorescence and biochemical studies showed that WspR clustering occurs at defined cellular locations during surface growth, producing localized c-di-GMP signaling rather than a uniform cytoplasmic increase [110]. Evidence suggests that the Wsp system detects changes in the cell envelope during surface engagement and that flagellar regulators influence WspR positioning and signaling output [111,112,113].

Mechanosensory input flows directly into c-di-GMP synthesis through *sadC*. The flagellar stator MotC contacts the transmembrane region of *sadC* and stimulates its catalytic activity. This interaction links flagellar load to the switch from motile to sessile growth [114]. Division of labor among DGCs is clear. RoeA channels c-di-GMP toward Pel production, whereas *sadC* has a stronger influence on the control of flagellar motility [115].

On the degradation side, the EAL domain PDE encoded by *bifA* regulates biofilm formation and swarming. Loss of *bifA* increases c-di-GMP and produces a hyperbiofilm and swarming-deficient state that can be restored by complementation [116]. RbdA, a PAS GGDEF EAL membrane protein, promotes dispersal. Structural analysis of its cytoplasmic region reveals an activated PDE dimer and coordinated movements between linked domains, which provide a mechanistic basis for PDE-driven dispersal [117,118]. Genome-wide surveys confirm that *P. aeruginosa* encodes many DGC and PDE proteins.

A defining feature of this network is the high proportion of dual-domain GGDEF–EAL enzymes. In most of these proteins, only one domain is catalytically active under physiological conditions, while the second domain serves a regulatory or sensory role [108]. Several of these enzymes are membrane-associated or show polar or clustered localization, supporting the concept of local c-di-GMP pools that selectively regulate motility, attachment, matrix production, or dispersal rather than global signaling [108]. These findings show that c-di-GMP control is modular and locally organized [119].

### 4.2. Effectors and Downstream Targets

Effector binding of c-di-GMP shifts matrix synthesis from permissive to fully active. PelD, a degenerate GGDEF domain receptor, binds dimeric c-di-GMP through an RXXD motif. Structural studies of PelD in its apo and bound states show that nucleotide binding activates Pel machinery and commits cells to Pel-dependent biofilm formation [120]. A parallel checkpoint regulates alginate synthesis. The inner membrane PilZ domain protein Alg44 requires c-di-GMP binding for alginate polymerization and export. Genetic work established *alg44* as essential for this activity, and structural studies confirm dimeric c-di-GMP as the activating ligand [74,107,121].

Furthermore, c-di-GMP also controls adhesin display through the LapD and LapG system. High c-di-GMP bound to LapD sequesters LapG and stabilizes surface adhesins. When c-di-GMP levels fall, LapG cleaves adhesins and reduces attachment. In *P. aeruginosa*, LapG cleaves the matrix adhesin CdrA, which fine-tunes aggregation and cohesion under c-di-GMP control [122,123,124]. Beyond matrix assembly and adhesion, c-di-GMP reshapes motility. High levels suppress flagellar motility and regulate type IV pili behavior during surface commitment. Mechanosensory coupling from the flagellar motor through MotC and *sadC* provides a direct path into c-di-GMP synthesis and consolidates the attached lifestyle [125,126].

### 4.3. Environmental and Host Signals Modulating c-di-GMP Turnover

Environmental and host-derived cues reset c-di-GMP levels and shift the balance among adhesion, matrix formation, motility, and dispersal. Low nonlethal NO activates PDEs, lowers c-di-GMP, and induces detachment. This mechanism is supported by biochemical studies, models that mimic the airway, and analyses of CF sputum. It is under clinical investigation as an adjunct to antibiotic therapy [72,127,128].

Temperature is another strong regulator. The thermosensory diguanylate cyclase A increases its catalytic rate more than one hundred-fold across a ten-degree change in temperature. This behavior tunes motility, biofilm development, and virulence. Similar thermosensory activity is reported in orthologs from other bacteria [129,130].

Cross-kingdom metabolites also shape lifestyle decisions. Ethanol produced by *Candida albicans* increases c-di-GMP in *P. aeruginosa*, inhibits swarming, strengthens Pel-dependent biofilms, and alters phenazine output. This response partly involves the Wsp system [131].

Oxygen limitation and related stresses feed into multisensor DGC and PDE proteins. PAS domain enzymes such as DipA and RbdA display higher PDE activity under nutrient or redox shifts. Increased PDE activity lowers c-di-GMP and triggers dispersal. ProE, a GGDEF–EAL hybrid protein, represses exopolysaccharide-related genes. These enzymes act as environmental sensors that couple oxygen and redox state to matrix-remodeling [117,132,133].

NO, temperature, ethanol, and hypoxia-related signals converge on c-di-GMP turnover and recalibrate the lifestyle of *P. aeruginosa* in clinical niches. These inputs either stabilize matrix-rich biofilms or prepare cells for dispersal [130,134].

### 4.4. Interplay with QS and Stress Pathways: Fine-Tuning Motility, EPS Synthesis, and Dispersal

c-di-GMP connects with QS and global regulators to coordinate the transition from acute to chronic states. LasR and RhlR promote matrix gene expression. For example, *psl* is under LasR and RhlR control. Higher c-di-GMP strengthens these QS-driven outputs and shifts cells toward matrix synthesis and away from motility [69]. This coordination sits within a larger network that links c-di-GMP, QS, cAMP Vfr, and Gac Rsm, aligning environmental information with virulence programs [68].

Posttranslational control further stabilizes outputs. Dimeric c-di-GMP binds Alg44 and activates alginate polymerization. AlgU drives *algD* expression and intersects with QS to promote mucoid behavior [74,135,136]. The Gac Rsm cascade shapes c-di-GMP flow and matrix-related genes. RsmA represses *pslA* and *sadC*, and the RetS and LadS pathways coordinate with DGCs such as *sadC* to bias populations toward either biofilm formation or motility [137,138]. The cAMP Vfr axis also interacts with c-di-GMP to reduce acute traits as chronic behavior rises [79].

**Figure 2:**
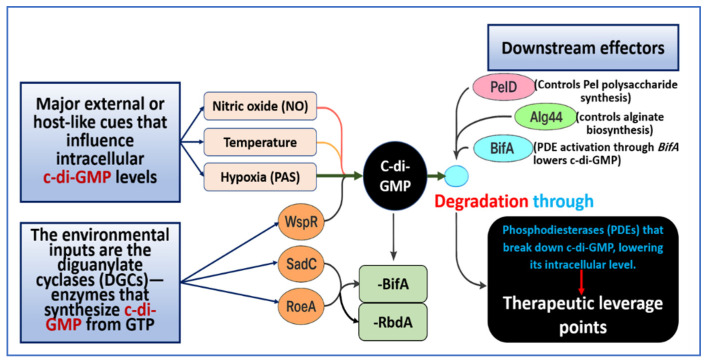
c-di-GMP network dynamics in *P. aeruginosa*. Environmental and host-related signals that include nitric oxide, temperature changes, and hypoxia or redox-linked cues sensed by PAS domain regulators converge on diguanylate cyclases that include WspR, *sadC*, and RoeA, and on phosphodiesterases such as *BifA* and RbdA. These enzymes set intracellular c-di-GMP levels and shape biofilm behavior. High c-di-GMP promotes matrix assembly. PelD activates Pel polysaccharide synthesis, and Alg44 promotes alginate polymerization. Low c-di-GMP, produced through PDE activity or nitric oxide-mediated reduction, favors motility and detachment. The schematic reads from left to right and shows inputs, enzymes, the c-di-GMP pool, and downstream effectors or phenotypes. The figure also highlights therapeutic intervention points at DGCs, PDEs, and c-di-GMP-dependent effectors. Abbreviations: DGC, diguanylate cyclase; PDE, phosphodiesterase; EPS, extracellular polymeric substance.

This network remains capable of reversal. Low nonlethal NO activates PDEs, reduces c-di-GMP, and initiates detachment. This principle supports the investigation of NO-based approaches in airway infections [21,139]. Figure 2 and Table 2 summarize this landscape. The figure maps environmental inputs, enzymes, and effectors. The table lists representative DGC and PDE proteins, their stimuli, dominant phenotypes, and structural insights. These elements highlight c-di-GMP as a central switchboard that links environmental cues and quorum signals to chronic biofilm behavior.

## 5. Antimicrobial Tolerance and Persistence of the Biofilm Code

### 5.1. EPS Mediated Diffusion Restriction and Charge Buffering

The matrix of *P. aeruginosa* functions as an active filter rather than a passive outer layer. Psl and Pel form the structural scaffold and slow the movement of antibiotics, which allows interior cells to survive longer. In early biofilms, Psl provides a broad protective barrier that limits the penetration of chemically diverse drugs and even shields nearby cells that do not produce Psl but occupy Psl-rich clusters [140]. Pel adds mechanical strength and charge-based interactions. Because Pel is a cationic polysaccharide, it crosslinks with negatively charged eDNA, tightens the network, and restricts diffusion. Structural and imaging studies demonstrate this ionic Pel and eDNA binding, and Pel can partially compensate when Psl is reduced, explaining why Pel-dominated biofilms maintain resilience [141].

Electrostatic effects deepen these defenses. eDNA is abundant in *P. aeruginosa* biofilms and binds cationic antibiotics, including aminoglycosides, which lowers their activity. Removing eDNA increases aminoglycoside killing [142,143]. Divalent cations, such as calcium, bridge eDNA strands and compact the matrix, while alginate-derived guluronate oligomers disrupt these cation bridges and loosen the network. This disruption increases antibiotic access to *P. aeruginosa* biofilms [144,145]. In clinical contexts, these matrix-based effects matter. In mature biofilms, colistin tolerance often reflects penetration limits and altered physiology rather than classical target site changes. These findings illustrate how diffusion and charge buffering establish a strong baseline of protection in vivo [146]. In summary, Psl slows drug entry at the perimeter, Pel and eDNA interactions strengthen internal layers, and eDNA and cation chemistry restrict cationic agents. Together, they create a barrier that routine drug dosing rarely overcomes.

### 5.2. Efflux Pumps and Stress Regulons

Efflux contributes a second defensive layer that aligns with the biofilm lifestyle. In mature biofilms, the transcriptional regulator BrlR increases tolerance by promoting expression of the RND pumps *mexAB-oprM* and *mexEF-oprN*, which reduce intracellular antibiotic concentrations even when diffusion barriers are partially bypassed [147,148]. Efflux is primed early in development. The sensor and regulator SagS function as a surface commitment factor and increase BrlR activity, which links early adaptation to activation of efflux before the matrix is complete [149].

In clinical isolates, these systems operate in combination. Large surveys show context-dependent coordination between *mexAB-oprM* and *mexEF-oprN*, with common co-expression alongside other RND pumps such as *mexXY* and *mexCD-oprJ* under stress and host-like cues [150]. Reviews consistently identify these two pumps as major contributors to *P. aeruginosa* resistance [81,151]. This has therapeutic implications. In flow-cell biofilms, colistin tolerance depends on both the PmrAB two-component system and *mexAB-oprM*, linking environmental stress directly to active efflux and explaining reduced activity of cationic peptides in vivo [146,152]. Efflux also influences virulence and fitness. Inhibiting pumps can reduce pathogenicity, and variants in *mexEF-oprN* alter motility and secretion pathways, demonstrating combined effects on drug survival and disease progression [153,154]. In summary, biofilm stage regulators such as SagS and BrlR coordinate *mexAB-oprM* and *mexEF-oprN* within a flexible efflux repertoire that, together with the matrix, creates a two-tier defense that many standard regimens fail to overcome [147,149,150].

### 5.3. Dormant, Persister, and VBNC Populations

Steep oxygen and nutrient gradients create microenvironments of slow-growing or non-growing cells that tolerate antibiotics without heritable resistance. Low oxygen interiors reduce the activity of bactericidal drugs and activate stress responses, so neighboring cells with the same genotype may differ markedly in susceptibility. This phenomenon helps explain incomplete killing during otherwise appropriate therapy [2,155,156]. Persisters emerge within this setting. These rare, phenotypically tolerant cells survive lethal antibiotic exposure and repopulate the community once treatment pressure declines. Across *P. aeruginosa* models, persisters contribute to relapse and chronicity and are supported by nutrient limitation, redox stress, and matrix protection [2,51]. Clinically relevant stresses also generate VBNC states. Sub-inhibitory tobramycin can induce and maintain VBNC physiology in biofilms, and chlorine exposure produces VBNC populations with distinct metabolic signatures. These transitions arise more frequently in biofilms than in planktonic cultures [54,157,158,159]. Together, persisters and VBNC cells form a deep reservoir of tolerance that is strengthened by matrix barriers and efflux. Conventional MIC assays overlook these features. More suitable metrics include the minimum biofilm inhibitory concentration (MBIC) and the minimum biofilm eradication concentration (MBEC), which better reflect biofilm antibiotic responses [2,160].

### 5.4. Horizontal Gene Transfer and Plasmid Stabilization Inside the Matrix

Biofilms provide strong support for gene exchange. Surface-attached growth improves physical contact among cells and stabilizes interactions, which increases conjugation frequencies relative to planktonic states. At the same time, spatial structure can restrict exchange to outer layers and create organized gene exchange zones [161,162]. Biofilms can also serve as plasmid reservoirs. Even without antibiotics and despite the burden of plasmid carriage, communities maintain plasmids and rapidly expand plasmid-containing populations when selection returns. Evolution experiments and microfluidic studies identify biofilms as important refuges for multidrug-resistant (MDR) plasmids, which shape transfer routes and explain the persistent presence of resistance genes in chronic infection [163,164].

Plasmid-encoded stabilization modules strengthen this persistence. Toxin and antitoxin loci and partitioning systems reduce plasmid loss during cell division and influence host fitness. These factors increase the likelihood that resistance and tolerance genes remain in the population [165,166]. In *P. aeruginosa*, mobile DNA circulates through multiple routes. Integrative and conjugative elements, such as PAPI 1 and outer membrane vesicles, transfer genetic material, including resistance determinants, which broaden adaptive pathways inside biofilms [167,168]. Because plasmid fitness effects depend on the host background and the surrounding environment, persistence becomes highly probable in biofilm-dominated niches [169].

MIC assays do not capture these realities. MIC measurements reflect planktonic inhibition under well-mixed conditions and do not account for diffusion barriers, charge buffering, oxygen gradients, efflux, or dormancy. As a result, MBIC and MBEC values often exceed MIC values by large margins. Biofilm-specific antimicrobial susceptibility testing (AST) platforms offer more accurate guidance for infections that involve devices or tissue surfaces and are increasingly recommended for translational studies [170,171,172,173].

**Figure 3:**
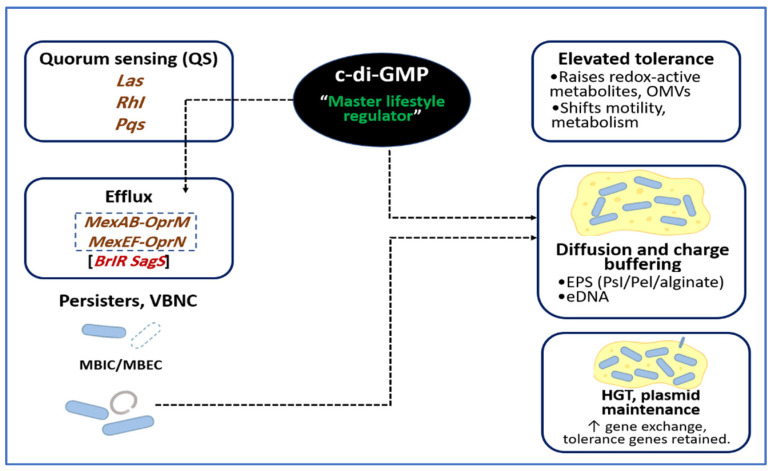
Quorum-sensing and c-di-GMP networks reinforce antimicrobial tolerance in *P. aeruginosa* biofilms. QS hierarchies that include Las, Rhl, and Pqs, together with the second messenger c-di-GMP, function as coordinated regulatory hubs that strengthen tolerance in biofilm communities. Elevated c-di-GMP acts as a master lifestyle regulator and promotes EPS synthesis through Psl, Pel, and alginate, and increases eDNA accumulation, which restricts antibiotic penetration and buffers cationic agents. In parallel, QS and c-di-GMP enhance the expression of efflux regulators *brlR* and *sagS,* and RND pumps *mexAB-oprM* and *mexEF-oprN*, which improve intracellular detoxification. These combined interactions favor the formation of persister and VBNC populations and help maintain horizontal gene transfer and plasmid stability within the matrix. Together, this layered regulatory architecture produces a biofilm tolerance phenotype in which MBIC and MBEC values rise well above conventional MIC thresholds.

In summary, the matrix functions as both a chemical shield and a genetic exchange zone. Spatial structure increases encounters, stabilization systems secure inheritance, and multiple transfer pathways support the dissemination of adaptive traits. These factors preserve resistance even when MIC-based therapy appears suitable [162,167,168]. Figure 3 illustrates how QS hierarchies and the c-di-GMP network coordinate tolerance in *P. aeruginosa* biofilms by guiding EPS production, activating efflux, supporting metabolic dormancy, and stabilizing plasmids.

## 6. Cracking the Code: Emerging Strategies to Rewire or Disrupt the Network

### 6.1. Quorum-Sensing Inhibitors (QSIs) and Quorum-Quenching Enzymes

Reducing QS signaling can quiet community behavior without applying strong growth pressure. In *P. aeruginosa*, synthetic halogenated furanones inspired by metabolites from *Delisea pulchra* antagonize AHL-mediated signaling. These compounds suppress Las-regulated transcription and reduce biofilm-associated virulence while leaving growth largely unchanged [174,175]. Ajoene, derived from garlic, offers an additional route. It decreases QS-regulated traits and alters the Hfq and small RNA layer that coordinates QS outputs. These effects reduce pathogenic behavior in vitro and in infection models [176,177]. Natural product discovery continues to expand the field. Methyl gallate from *Mangifera indica* lowers QS-linked outputs that include pyocyanin, rhamnolipids, and motility at sub-MIC levels, and docking studies support LasR engagement together with broad antivirulence activity [178,179].

Enzymatic quorum quenching removes the signal directly. The periplasmic acylase PvdQ hydrolyzes long-chain AHLs involved in Las signaling. Structural work has mapped its active site, and administration of PvdQ in a murine lung model reduced inflammation and bacterial burden. Protein engineering now improves stability and catalytic performance [180,181,182]. Other AHL lactonases and acylases from diverse microbes also reduce QS-dependent virulence and biofilm formation and are being refined as adjuncts to antibiotics [183]. CRISPR interference provides another layer. Silencing of *lasI*, *rhlI*, and *pqsR* has been demonstrated in vitro and in vivo, and recent work connects QS, cAMP Vfr, and CRISPR systems, broadening antivirulence strategies [184,185,186,187]. By reducing Las, Rhl, and Pqs activity or degrading their autoinducers, QS inhibitors and quorum-quenching enzymes limit biofilm formation and virulence and often restore antibiotic susceptibility.

### 6.2. c-di-GMP Modulators and Dispersal Triggers

Reducing intracellular c-di-GMP can open established biofilms. Small molecules that activate native PDEs or inhibit DGCs shift cells from sessile to motile states and briefly increase antibiotic susceptibility. An example is the H6-335 chemotype. In *P. aeruginosa*, it reduced c-di-GMP, prevented biofilm formation, and dispersed established biofilms. An analog, H6-335-P1, activated the *BifA* PDE and, when combined with antibiotics, cleared implant-associated infections in mice [188,189].

NO remains the most validated physiological dispersal trigger. At low concentrations, it activates PDEs that target c-di-GMP, lowers intracellular c-di-GMP, and releases cells from the matrix. Dispersal depends on PDE activity in flow-cell models [21,190,191]. Host-derived cues can induce similar shifts. The mouse cathelicidin CRAMP triggers dispersal with associated changes in c-di-GMP regulators [192]. Combining a c-di-GMP-lowering compound with an appropriate antibiotic collapses biomass and increases killing. This approach is supported by mechanistic data on PDEs such as RbdA and by the in vivo results [117,118].

### 6.3. Nanoparticle-Based Synergistic Systems

NPs provide versatile platforms to disrupt *P. aeruginosa* biofilms by improving drug penetration, generating localized stress, and co-delivering agents that weaken tolerance networks. Surveys catalog metal and metal-oxide particles that include silver (Ag), zinc oxide (ZnO), and titanium dioxide (TiO_2_), as well as mesoporous silica frameworks, polymer and lipid carriers, and hybrid systems that restore antibiotic activity at lower doses [193,194].

Metal-based systems deliver broad stress. AgNPs damage membranes, respiratory enzymes, and nucleic acids and act synergistically with standard antibiotics against MDR *P. aeruginosa*. Recent reviews outline proteomic stress responses and dosing strategies that balance antimicrobial activity with host safety [195,196]. ZnONPs and TiO_2_NPs can generate reactive oxygen species under defined conditions and display antibiofilm and antibiotic potentiating activity. Very low zinc oxide can be hormetic, which underscores the need for controlled exposure [197,198]. Photocatalytic TiO_2_ coatings are being developed as self-cleaning and antibiofilm surfaces for devices and filters [199].

Mesoporous silica NPs (MSNPs) allow high drug loading and tunable release. Hybrid silica and gold nanomotors activated by near infrared (NIR) light penetrate biofilms and reduce biomass through photothermal and photocatalytic effects within short periods [200]. Silica-based NO-releasing particles kill biofilm cells and enhance antibiotic activity, confirming NO as a programmable dispersal and killing cue at the nanoscale [201]. Design studies describe mesoporous silica systems that couple antimicrobial drugs with stimuli-responsive release based on light, pH, or redox conditions [202].

In the airway, liposomal formulations improve residence time, reduce epithelial toxicity, and enhance penetration of mucus-rich and matrix-rich regions. In a chronic *Pseudomonas* rat model, liposomal bismuth ethanedithiol lowered bacterial counts. Reviews support inhaled, NP enabled antibiotic strategies for difficult lung infections [203,204,205]. Co-delivery of antibiotics with QS antagonists or c-di-GMP modulators aims to weaken multiple tolerance layers at once. Proof-of-concept work shows gold and selenium-based NPs reduce QS-controlled outputs such as pyocyanin and elastase, while design papers discuss gene targeting approaches and exposure controls to minimize environmental effects [206,207]. Dose, irradiation settings, and matrix interactions strongly shape outcomes. ZnO hormesis highlights the need for therapeutic windows and standardized biofilm testing that includes MBIC and MBEC alongside MIC [198]. By uniting targeted delivery with microenvironment-responsive release and combining antibiotics with QS or c-di-GMP inhibitors, NP systems penetrate EPS, promote dispersal, and increase susceptibility of entrenched biofilms [200,201,208].

### 6.4. Phage and Phage-Derived Enzymes

Bacteriophages offer precision tools for dismantling *P. aeruginosa* biofilms by damaging both matrix and cells. Native or engineered phages equipped with polysaccharide depolymerases degrade EPS and expose embedded cells to antibiotics and immune factors. Reviews document strong biofilm-degrading activity against Gram-negative biofilms, including *P. aeruginosa* [209,210].

Endolysins and next-generation Artilysins, which fuse lysins with peptides that permeabilize the outer membrane, can lyse *P. aeruginosa* despite the Gram-negative barrier. Rationally designed constructs, including LysPA26-based hybrids, show strong antibiofilm activity [211,212]. Phage antibiotic combinations often outperform either alone. In vitro and in vivo models that include zebrafish and murine airway and implant systems show accelerated clearance and improved survival, with reduced emergence of resistance [213,214,215].

Directed evolution to improve penetration and cocktail design to exploit resistance tradeoffs can strengthen performance against persistent biofilms [216]. The endogenous glycoside hydrolase (PslG) functions as a phage enzyme-like adjunct. Applied exogenously, it disperses Psl-rich biofilms and increases antibiotic susceptibility [217,218,219]. Phage, depolymerase, and engineered lysin strategies therefore provide targeted matrix digestion and bacterial killing that complement chemical inhibitors and antibiotics [220].

### 6.5. Host-Directed and Immunomodulatory Therapies

Because *P. aeruginosa* biofilms evade immune clearance, host-focused therapies are emerging alongside antibacterial agents. The bispecific monoclonal antibody MEDI3902, also known as gremubamab, targets Psl and the type three secretion protein PcrV. Phase one studies show favorable safety and pharmacokinetics (PKs) in healthy adults, and preclinical models show protection in bacteremia and pneumonia. Broad target conservation supports clinical applicability [221,222,223].

Vaccine development is active. Foundational work on alginate conjugates and the OprF and OprI formulation IC43 shaped the field. Newer strategies, such as NP presented antigens and multi-epitope constructs, aim to elicit strong antibody responses against adhesins and secretion components relevant to chronic lung disease [224,225,226]. Matrix targeting biologics complements these approaches. Enzymes directed at Psl, including variants of PslG, degrade EPS and increase antibiotic and neutrophil access, which improves host clearance [217,219]. These therapies are increasingly positioned as adjuncts combined with antibiotics, QS or c-di-GMP modulators, or phage to favor host control [224].

Host-directed therapies counter adhesins and secretion systems, generate protective antibodies, and weaken the matrix. These functions shift treatment from antibiotic killing alone to immune-enabled biofilm control, supported by clinical-stage antibodies and next-generation vaccine candidates [221,222].

**Figure 4:**
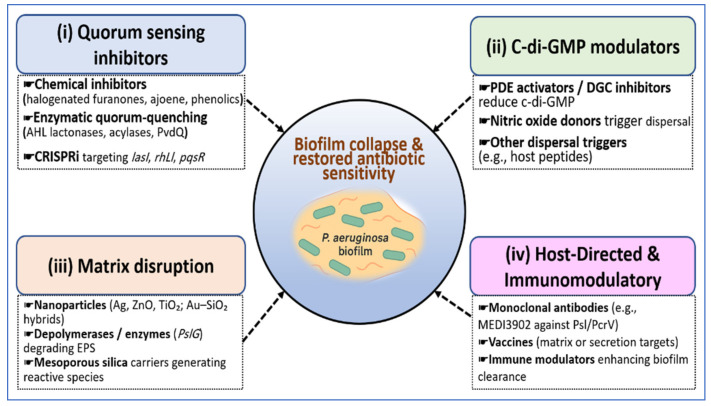
Mechanistic overview of network disruption strategies targeting the *P. aeruginosa* biofilm code. Four complementary therapeutic modules converge to dismantle biofilms and resensitize bacteria to antibiotics. Quorum-sensing inhibition uses small-molecule antagonists, signal-degrading enzymes, and CRISPR interference that targets *lasI*, *rhlI*, and *pqsR* to mute virulence and community signaling. Furthermore, c-di-GMP modulators, including PDE activators, DGC inhibitors, and nitric oxide donors, lower intracellular c-di-GMP and promote dispersal with a shift toward motile states. Matrix disruption relies on nanoparticles, depolymerases such as PslG, and mesoporous silica carriers to degrade EPS and improve antibiotic penetration. Host-directed and immunomodulatory approaches employ monoclonal antibodies such as MEDI3902, vaccines, and immune modulators to enhance clearance. Together, these strategies collapse the biofilm tolerance architecture and restore the effectiveness of conventional antibiotics.

Table 3 outlines the major therapeutic tracks that include QS disruption, quorum-quenching enzymes such as PvdQ, CRISPR interference of *lasI*, *rhlI*, and *pqsR*, c-di-GMP-reducing agents such as H6-335, NO donors, matrix-focused NP platforms, phage and depolymerase combinations, and host-directed biologics such as MEDI3902. The table links each approach with its molecular target, model system, antibiofilm readouts, and translational status. Figure 4 places these interventions within four major control hubs that include QS, c-di-GMP signaling, matrix integrity, and host immunity and shows how they converge on biofilm collapse and antibiotic resensitization. Together, these strategies provide a system-level view of biofilm dismantling that aims to weaken coordination, reduce tolerance, and restore therapeutic activity.

## 7. Diagnostic and Analytical Horizons: Reading the Biofilm Code

### 7.1. Advanced Imaging: CLSM, OCT, and Raman Mapping as a Spatial Code

Confocal laser scanning microscopy (CLSM) remains a central method for tracking *P. aeruginosa* biofilm development, stress responses, and regression. It provides three-dimensional information on volume, live and dead ratios, height, surface coverage, and roughness. Recent progress lies in standardized analysis. Harmonized workflows reduce operator bias, which is critical when evaluating QS inhibitors or c-di-GMP ligands [237,238]. Benchmark studies that compare dyes and software tools show that consistent image acquisition combined with COMSTAT-based quantification improves estimates of biovolume, secondary thickness, and viability in *P. aeruginosa* single species and mixed species communities [238,239].

Optical coherence tomography (OCT) covers a mesoscopic range. It is label-free, depth-resolved, and rapid. OCT supports the real-time monitoring of biomass, void fraction and porosity, roughness, and shear-induced detachment on medical materials and industrial lines. Bioluminescence platforms can be paired with OCT to obtain structure-linked viable load readouts or machine learning (ML) based texture scoring over time. Mid infrared (mid IR) and frequency domain OCT (FD OCT) provide better contrast in soft, water-rich materials [240,241,242]. From early capillary flow imaging to current OCT and bioluminescence combinations, studies show that OCT enables rapid and nondestructive measurements of thickness, roughness, void distribution, and detachment on device-scale materials in both laboratory and field settings, including irrigation tubing and medical polymers [240,243,244,245].

Raman microspectroscopy, including surface-enhanced Raman spectroscopy (SERS) and confocal Raman mapping, measures matrix chemistry in situ. In *P. aeruginosa*, SERS and confocal Raman imaging detect phenazines such as pyocyanin and alkyl quinolones through depth. These approaches visualize quorum signal gradients and redox changes as antibiotics diffuse [246,247,248]. A recent study generated three-dimensional chemical maps with better depth resolution and clearer metabolite-rich layers [249]. Method papers now recommend combining label-free Raman maps with mass spectrometry imaging (MSI), including matrix-assisted laser desorption ionization (MALDI) MSI, to co-register structure and metabolite patterns and reveal the small-molecule code that underlies biofilm behavior [246,250,251]. Practical SERS, with validation by electrochemical SERS, tracks evolving gradients of virulence factors over time [246,252]. MALDI guided MSI workflows identify phenazines and other metabolites across depth, allowing true overlays of chemotypes and structure [253,254,255]. In practice, CLSM provides biovolume, live and dead ratios, surface coverage, and roughness. OCT offers thickness, porosity, and void fraction, and detachment kinetics. Raman and SERS supply depth-resolved maps of pyocyanin, HHQ, PQS, redox state, and antibiotic penetration [238,243,246].

### 7.2. Omics Integration: Transcriptomic and Proteomic Mapping of QS and c-di-GMP Signatures

Time-resolved dual omics approaches now monitor *P. aeruginosa* interactions with airway epithelial cells in real time. Dual RNA sequencing (RNA-seq) in organoids and air–liquid interface (ALI) cultures measures both host and bacterial transcripts during biofilm development and remodeling [256,257,258]. These datasets show that the QS hierarchy is conditional. Las, Rhl, and Pqs can reorder under different stresses and media conditions, which agrees with recent reinterpretations of QS regulation [61]. Integrated work supports a simple principle. Environmental cues reshape local c-di-GMP signaling and shift cells toward motile or matrix-dwelling states [259]. Biochemical studies link selected PDEs, including *rmcA* and *morA*, to maintenance of mature architecture and tolerance. These findings connect second messenger balance directly to structure and resilience [260].

Genome-scale perturbation is refining the regulatory map. Multiplex CRISPR editing and CRISPR interference across GGDEF and EAL enzymes reveal how distributed enzyme activity sets c-di-GMP levels and defines distinct biofilm and virulence phenotypes [261,262]. These results support a model in which c-di-GMP acts through local hubs and protein interaction networks rather than a single global control [263,264]. Proteomic studies are also identifying actionable markers. For example, PA2146 marks biofilm formation on endoscope channel polymers and demonstrates MALDI mass spectrometry as a useful monitoring tool [265]. The most informative pipeline now integrates RNA-seq and proteomics with structured imaging such as CLSM and OCT, and chemical maps from Raman and MSI. This combination allows QS and c-di-GMP nodes to be aligned with phenazine and PQS gradients and with physical morphology [253,266].

### 7.3. Rapid Diagnostics: MALDI-TOF Biofilm Profiling, Impedance Biosensing, and Microfluidic Biofilm on Chip Models

Beyond species-level identification, emerging proteomic methods support a biofilm stage marker panel. A distinct approximately 5.45 kilodalton MALDI time of flight (MALDI-TOF) feature corresponding to PA2146 increases during *P. aeruginosa* biofilm growth on biopsy channel polymers. This marker is validated by liquid chromatography tandem mass spectrometry and is absent in strains lacking the gene, suggesting a practical monitoring signal for endoscope reprocessing [265]. Wider surveys propose that MALDI-TOF combined with MALDI MSI can generate small-molecule fingerprints that reflect quorum regulation and matrix development. These studies also note the current lack of a general biofilm producer database across ESKAPE pathogens [267].

Microfabricated electrochemical impedance spectroscopy (EIS) devices track early attachment and progression to mature biofilm in near real time, often within the first hour. They generate multiphase spectra that correlate with biomass and matrix formation and support closed-loop testing of dispersal triggers or QSIs [268]. Newer architectures enlarge the electroactive area and incorporate flow, which yields large and reproducible impedance changes during growth and after induced dispersal. These advances support compact and inexpensive sensors for monitoring hospital waterlines and device surfaces [269,270]. Reviews of electrochemical biosensors integrated into microfluidic systems highlight high sensitivity, nondestructive measurement, and true time series tracking of biofilm behavior [271].

Standardized biofilm on chip platforms now enable MBIC and MBEC testing under flow, straightforward polymicrobial coculture, and well-controlled gradients that better resemble clinical niches [272]. CF airway organ chips add mucus viscosity, shear, and epithelial barriers and provide a pharmacodynamic (PD) context for antibiotics, QSIs, and c-di-GMP modulators under patient-relevant conditions [273]. A new direction combines MALDI guided MSI with chip-grown *P. aeruginosa* to analyze quorum signals, phenazines, and structure in parallel [254,274]. This supports a same-day detect, stage, and treat concept. MALDI-TOF markers such as PA2146 report on biofilm stage, EIS defines kinetics and spread, and microfluidic chips provide rapid MBIC and MBEC data to guide therapy [265,268,272].

### 7.4. AI and Systems Biology: Predicting Biofilm Phenotypes and QSI Efficacy with ML

Explainable machine learning (ML) models now predict *P. aeruginosa* resistomes and biofilm vulnerabilities from genomic or integrated omics data. These tools include interfaces that validate phenotype predictions, such as responses to tobramycin in chip-grown biofilms [275,276]. Clinical evaluations favor interpretable approaches such as Bayesian and logistic models, while feature-weighted gradient boosting incorporates molecular diagnostic inputs [277]. For antivirulence discovery, quantitative structure-activity relationship (QSAR) models combined with ML help prioritize candidate LasR and RhlR ligands or peptides before testing in biofilm on chip systems [271].

Network-centered studies confirm that c-di-GMP operates through localized hubs. Recent mapping work defines protein neighborhoods that predict sessile versus motile outcomes. These patterns align with CRISPR-based perturbation screens across GGDEF and EAL enzymes and highlight targets for combined QSI and PDE activator strategies [261,263]. Clinically, NO-triggered dispersal remains the best validated PDE activation approach. It transiently lowers c-di-GMP and enhances antibiotic activity, and is supported by controlled CF trials and progress in donor chemistry and delivery [134,278].

A proposed pipeline links these tools into a single workflow. First, OCT and CLSM provide morphometric data. Second, Raman and SERS define metabolite layers. Third, depth fractionated RNA-seq and proteomics reveal transcriptional and protein-level signatures. Fourth, models such as XGBoost and long short-term memory (LSTM) networks predict MBIC, MBEC, and dispersal probability under QS inhibitors and c-di-GMP modulators. Fifth, prospective tests on microfluidic chips with EIS readouts validate these predictions [268,271,272]. This integration moves biofilm assessment toward predictive and mechanism-aware diagnostics.

## 8. Challenges, Translational Barriers, and One Health Perspectives

Moving antibiofilm strategies toward clinical use requires attention to safety, regulatory expectations, and ecological impact. For NPs and enzyme-based therapies, safety assessment begins with biocompatibility standards. ISO 10993 guides this process. Part 1 outlines test selection, Part 5 addresses in vitro cytotoxicity, and recent guidance from the United States Food and Drug Administration provides conditions under which chemical data or literature can replace animal testing [279,280,281]. Nanomaterials require additional scrutiny. ISO Technical Report 10993-22 calls for explicit characterization of particle size, shape, agglomeration, surface chemistry, and dissolution. It also notes that common cytotoxicity assays may behave differently at the nanoscale [282]. Interlaboratory comparisons reveal substantial variability in ISO 10993-5 cytotoxicity outcomes. These findings highlight the need to standardize extraction conditions, cell lines, and readouts when evaluating candidate coatings or nanoparticle payloads [283,284,285]. Best practice is clear: define the material, document exposure, match device relevant conditions, and pair biological tests with chemical characterization using ISO 10993-18, so that any safety signal can be interpreted accurately [286].

None of these approaches prevents evolution. *P. aeruginosa* can develop reduced susceptibility to QSIs. Although antivirulence pressure may select more slowly than antibiotic pressure, reviews argue for combination strategies to slow escape [287,288,289]. Phage-based therapies face similar constraints. Cocktails designed for biofilms that target different receptors and combine complementary activities reduce escape routes, and evolution in biofilm assays and phage antibiotic combinations improve durability. Even so, resistance can emerge when dosing or local ecology is not aligned with treatment design [216,290]. Clinically, NO-based dispersal remains a practical adjunct. It can resensitize biofilm aggregates, and early trials and translational reviews outline guidance on donor selection, delivery methods, and endpoints [134,291,292].

A One Health perspective shows why translation must extend beyond patient care. *P. aeruginosa* persists in premise plumbing. Sink drains, U bends, and faucet components act as long-term reservoirs that seed patient rooms. Whole genome studies repeatedly link isolates from patients to drains and identify high-risk clones such as ST111 and ST235, as well as carbapenemase-producing strains in hospital water systems [293,294,295]. Chlorine-tolerant *P. aeruginosa* and antimicrobial resistance (AMR) plasmids within plumbing biofilms help explain why routine disinfection may fail without engineering controls and targeted remediation [296,297]. Outside hospitals, drinking water plumbing is recognized as an AMR hot spot. Low flow, stagnation, and limited disinfectant residuals support opportunistic pathogens and increase the potential for resistance exchange [298,299,300]. Global reports emphasize the scale of the problem. Resistance in Gram-negative pathogens continues to rise in hospitals worldwide. These trends argue for diagnostics and interventions that are validated not only in laboratory and animal models, but also in the water systems where biofilms persist [301,302].

## 9. Conclusions

*P. aeruginosa* biofilms are dynamic and adaptive systems that are coordinated by QS and c-di-GMP. These networks build a matrix, limit antibiotic access, and support tolerant phenotypes that survive treatment. Understanding this biofilm code clarifies why standard MIC testing often fails in chronic lung disease, wounds, and device-related infections. Limited penetration, active efflux, and persister and VBNC populations act together to protect the community. A practical therapeutic sequence is to disperse, then attack, then clear. First, lowering c-di-GMP and damping quorum signals opens the matrix and releases cells. Next, targeted antimicrobials reach previously protected populations. Finally, host-directed therapies or matrix-degrading adjuncts remove residual biomass. The available toolkit includes c-di-GMP modulators, NO-based dispersal strategies, QS antagonists, signal-degrading enzymes, NPs, enzymatic depolymerases such as Psl targeting PslG, bacteriophage cocktails, and clinical-stage antibodies that recognize Psl or PcrV. Biofilm-aware susceptibility testing through MBIC and MBEC should guide treatment choices rather than planktonic MIC values. Successful translation requires safety by design that is informed by standards such as ISO 10993 for materials and nanosystems, together with standardized and clinically relevant diagnostics. A One Health perspective is also essential, with attention given to reservoirs in both patients and infrastructure. With mechanism-guided combinations, realistic biofilm testing, and upstream surveillance, dismantling the *P. aeruginosa* biofilm problem becomes an achievable goal.

## Figures and Tables

**Table 1:** QS systems and regulatory integration in *P. aeruginosa*.

QS System	Signal(s)	Synthase/Receptor	Major Regulatory Links	Principal Outputs/Traits	Environmental Modulators	Representative Drug Targets	Reference
Las	3 oxo C12 HSL	LasI and LasR	Activates *rhl* and *pqs* and functions as a central regulator in the QS hierarchy	Elastase (*lasB*) and proteases, exotoxin A, and initiation of biofilm formation	Nutrient sufficiency and oxygen availability	LasR antagonists and LasI inhibitors	[16,102]
Rhl	C4 HSL	RhlI and RhlR	Represses *pqsABCDE* during phosphate stress and forms a functional partnership with PqsE	Rhamnolipids (*rhlAB*) and hydrogen cyanide, and control of motility	Phosphate limitation	RhlR antagonists and compounds that disrupt the RhlR and PqsE interaction	[60,65]
Pqs (MvfR)	HHQ and PQS	*PqsABCDH* and PqsR (MvfR)	Regulates phenazine production as well as eDNA and OMV release, and provides feedback to QS	Pyocyanin and other phenazines and eDNA, OMVs, and promotion of dispersal	Iron availability and redox-related stress	PqsR antagonists and inhibitors of PQS biosynthesis	[103,104,105]
Iqs	IQS	*AmbBCDE* and IqsR are responsive to PhoB	Links phosphate stress through PhoB to activation of *rhl* and *pqs*	Stress-responsive activation of virulence programs	Low phosphate	Inhibitors of the PhoB IQS signaling pathway	[61,106]
Global integration	Multiple	RhlR with PqsE and AlgU with Alg44 and c-di-GMP and Gac and Rsm and Vfr	Coordinates QS and c-di-GMP cross-talk and Alg44 activation and higher-level regulation	Expression of *psl, pel*, and alginate, and formation of chronic biofilms and persistence	Host-related stresses and mucoid conditions	Enzymes that control c-di-GMP and modulators of AlgU and Alg44	[18,74,107]

**Table 2:** Representative c-di-GMP enzymes in *P. aeruginosa* inputs, phenotypes, and mechanisms.

Enzyme (Protein/Gene)	Domain Architecture	Primary Input/Stimulus	Dominant Phenotype	Mechanism/Notes	Reference
WspR (*wspR*)	GGDEF response regulator DGC	Surface engagement through WspA and WspE and cell envelope perturbation	Increased biofilm formation and reduced motility	Phosphorylation-dependent subcellular clustering enhances DGC activity	[111,112]
SadC (*sadC*)	Membrane-associated GGDEF	Flagellar stator load through MotC interaction	Reduced motility and increased biofilm formation	MotC engages sadC to stimulate c-di-GMP synthesis	[114]
RoeA (*roeA*)	Inner membrane GGDEF	Control of matrix production	Increased Pel-related EPS synthesis	Channels c-di-GMP to Pel machinery and functions separately from *sadC* control of motility	[34,115]
TdcA (*tdcA*)	GGDEF thermosensory DGC	Temperature increase at a strong rate across a ten-degree range	Temperature-dependent motility, biofilm formation, and virulence	Thermosensory activity of TdcA produces marked increases in c-di-GMP with warming	[130]
BifA (*bifA*)	EAL domain PDE	Homeostatic degradation of c-di-GMP	Active *BifA* supports lower biofilm levels and greater swarming motility	Deletion of *bifA* elevates c-di-GMP and produces a hyperbiofilm and poor swarming state	[116]
RbdA (*rbdA*)	PAS GGDEF EAL PDE	Redox and oxygen-linked signals	Increased dispersal through PDE activation	Allosteric activation of the EAL domain by GTP binding to the GGDEF domain	[117]
DipA (*dipA*)	Multidomain EAL PDE	Nutrient shifts and dispersion cues	Increased dispersal that depends on PDE activity	Essential for induced dispersion with rising PDE activity during dispersal	[33,132]
ProE (*proE*)	GGDEF EAL hybrid with active PDE	Redox and nutrient inputs with local EPS control	Restraint of the EPS gene expression when active	Highly active PDE that suppresses EPS transcription and shows polar localization	[133]

**Table 3:** Therapeutic modalities targeting *P. aeruginosa* biofilms: mechanisms, models, outcomes, and translational status.

Modality	Molecular Target(s) andMechanism	Representative Agent(s)	Primary Model(s) Tested	Key Antibiofilm Outcomes	Translational Readiness	Reference
QSIs	Antagonize AHL-mediated QS through Las and Rhl; reduce QS-regulated virulence and EPS without strong growth inhibition	Halogenated furanones; ajoene	In vitro flow cells; mammalian infection models	Reduced Las-controlled transcription, lower pyocyanin and rhamnolipids, decreased biofilm-associated virulence at sub-MIC levels	Lead stage with in vivo proof-of-concept	[174,176]
Natural QS antagonists	Inhibit LasR and RhlR activation and downstream QS outputs	Methyl gallate	In vitro multi-strain *P. aeruginosa* panels; docking and biochemical assays	Reduced QS phenotypes, including pyocyanin, motility, and rhamnolipids, at sub-MIC levels	Preclinical discovery and optimization	[178,227]
Quorum quenching enzymes	Enzymatic degradation of AHL signals	PvdQ acylase; AHL lactonases and acylases	Murine lung infection models; in vitro biofilms	Attenuated QS activity and decreased biofilm burden; adjuvant potential with antibiotics	Enzyme engineering in progress	[180,181,228]
CRISPRi against QS nodes	Programmable repression of *lasI*, *rhlI*, and *pqsR*	dCas9-based CRISPRi constructs	In vitro systems; emerging in vivo models	Targeted QS knockdown with reduced virulence and biofilm formation	Platform stage	[187,229]
c-di-GMP modulators	Lower c-di-GMP through PDE activation or DGC inhibition; promotes dispersal	H6-335 class; H6-335-P1	In vitro biofilms; murine implant infection models	Dispersal of established biofilms and enhanced clearance with antibiotics	Strong preclinical signal	[188]
NO-based dispersal triggers	Activate PDEs and transiently reduce c-di-GMP	Low-dose NO donors	Flow-cell and airway models	Rapid dispersal and increased antibiotic susceptibility	Adjunct concept with growing clinical interest	[21,230]
Metal/metal-oxide NPs	Generate reactive species, disrupt membranes and proteins, enhance penetration	AgNPs; ZnO; TiO_2_	In vitro biofilms; device coatings; preclinical studies	Synergy with antibiotics; antibiofilm surface formation	Variable; dose and irradiation windows critical	[231,232]
Phages with depolymerases	Matrix digestion and lytic killing; improve penetration	Phage cocktails; polysaccharide depolymerases	In vitro, zebrafish, murine airway/implant models	Strong biofilm reduction; synergy with antibiotics	Advancing through preclinical development	[213,233,234,235]
Monoclonal antibodies	Neutralize adhesins and secretion components; enhance opsonophagocytosis	MEDI3902 (anti-Psl and anti-PcrV)	Phase 1 studies; preclinical pneumonia/bacteremia models	Established safety and pharmacokinetics; protective effects	Clinical-stage monoclonal antibody	[221,236]

## Data Availability

No new data were created or analyzed in this study. Data sharing is not applicable to this article.
